# Extracellular vesicle associated and soluble immune marker profiles of psychoneurological symptom clusters in men with prostate cancer: an exploratory study

**DOI:** 10.1038/s41398-021-01554-w

**Published:** 2021-08-24

**Authors:** Dilorom Sass, Leorey Saligan, Wendy Fitzgerald, Ann M. Berger, Isaias Torres, Jennifer J. Barb, Kevin Kupzyk, Leonid Margolis

**Affiliations:** 1 National Institute of Nursing Research, National Institutes of Health, Bethesda, MD USA; 2 University of Nebraska Medical Center, Omaha, 68105 NE USA; 3 Section on Intercellular Interactions, Eunice Kennedy-Shriver National Institute of Child Health and Human Development, National Institutes of Health, Bethesda, MD USA; 4 Clinical Center, National Institutes of Health, Bethesda, MD USA

**Keywords:** Biomarkers, Pathogenesis

## Abstract

Psychoneurological symptom clusters are co-occurring and interrelated physiological symptoms that may include cancer-related fatigue, pain, depressive symptoms, cognitive disturbances, and sleep disturbances. These symptoms are hypothesized to share a common systemic proinflammatory etiology. Thus, an investigation of systemic immune biomarkers is an important approach to test this hypothesis. Here, we investigated the associations between extracellular vesicle (EV)-associated and soluble cytokines with immune markers and symptom clusters in men with non-metastatic prostate cancer. This observational study included 40 men with non-metastatic prostate cancer at the start (T1) of external beam radiation therapy (EBRT) and 3 months post treatment (T2), as well as 20 men with non-metastatic prostate cancer on active surveillance (AS) seen at one time point. Collected questionnaires assessed patient-reported fatigue, sleep disturbances, depressive symptoms, and cognitive fatigue. In total, 45 soluble and EV-associated biomarkers in plasma were determined by multiplex assays. Principal component analysis (PCA) was used to identify psychoneurological symptom clusters for each study group and their time points. Bivariate correlation analysis was run for each identified PCA cluster with the concentrations of EV-associated and soluble cytokines and immune markers. Both EV-associated and soluble forms of RANTES significantly correlated with the symptom cluster for EBRT at T1, whereas, at T2, soluble IFNα2, IL-9, and IL-17 correlated with the corresponding symptom cluster. For the AS group, soluble survivin correlated with psychoneurological symptoms. Linking specific inflammatory cytokines with psychoneurological symptom clusters in men receiving prostate cancer treatment can enhance understanding of the underlying mechanisms of this phenomenon and aid in developing targeted interventions.

## Introduction

Cancer survivors often face many co-occurring, psychoneurological symptoms such as cancer-related fatigue, pain, depression, sleep disturbances, and cognitive impairments [1–3] that can lead to poor health-related quality of life [4]. These interrelated symptoms form clusters that seem to be related to psychological and/or neurological dysfunction [5] in patients with cancer.

Multiple studies connected psychoneurological symptom clusters to inflammation, activation of sympathetic nervous system, and activation of hypothalamic–pituitary–adrenal (HPA) axis) [2]. Previously identified cytokine gene polymorphisms in patients with higher symptom burden include C-reactive protein (CRP), interleukin 4 (IL-4), IL-6, IL-7, and soluble tumor necrosis factor receptor 1 (sTNF-R1) [2]. It has been hypothesized that peripheral inflammation affects these symptoms through the expression of cytokines within the brain triggering sickness-like behavior, including fatigue and depression [6, 7].

In patients with cancer treated with cytokines (e.g. IL-2, IL-2 +IFNα, and IFNα), 50% of subjects developed mild depressive symptomology and 22% developed moderate to marked depressive symptomology. Moreover, cytokine administration led to the development of neurovegetative symptoms (decreased sleep and appetite), cognitive disturbances, and emotional affective symptoms (sadness, inner tension) [8]. In another study, in patients with non-small lung cancer treated with epidermal growth factor receptor tyrosine kinase inhibitor, the circulating levels of proinflammatory chemokine, RANTES, was significantly associated with increased severity of general fatigue [9]. In contrast, in patients with psoriasis, injections of TNF-blocking agent, Entracept, resulted in reduced fatigue and depressive symptoms [10]. These studies affirm that inflammatory cytokines play a role in expression of psychoneurological symptoms.

Clinically, it is challenging to draw clear connections between peripheral cytokines and the brain; thus, peripheral markers that are able to cross blood–brain barrier can be informative in relation to behavioral outcomes. Extracellular vesicles (EVs) could serve as markers since it was shown that they can cross the blood–brain barrier and carry inflammatory and neurogenerative molecules from periphery to the brain and vice versa [11].

**Fig. 1:**
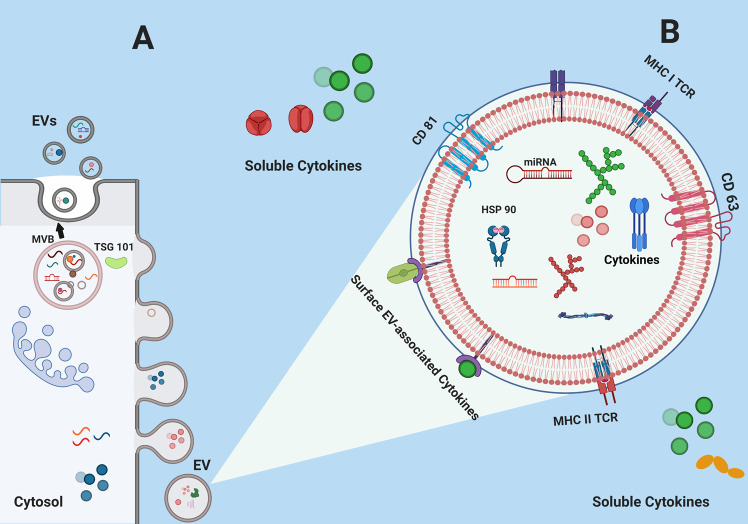
Illustration of extracellular vesicles (EV). **A**. Release of EVs from multivesicular body (MVB); **B** EV with common genetic components and proteins including cytokines [12, 13].

EVs (30–1000 nm in diameter) produced by cells of many types carry tetraspanins (CD9, CD63, CD81, CD82), heat-shock proteins (HSP70, HSP90), cytokines, miRNAs, MHC class I and MHC class II, and many proteins [12, 13]. Figure 1A illustrates release of EVs from a multivesicular body and Fig. 1B illustrates an enlarged image of an EV with previously described common genetic and protein markers [12, 13].

Earlier, it was demonstrated that cytokines are present in EV-associated and free (soluble) forms in a number of in vitro, ex vivo, and in vivo systems and both forms of the cytokines are biologically active [14]. Investigation of EV-associated cytokines relations to the psychoneurological symptoms may reveal their role as behavioral mediators. To our knowledge, such an investigation has not been previously undertaken. Therefore, the aim of our exploratory study was to investigate the associations of EV-associated and soluble cytokines, chemokines, and heat-shock proteins in two cohorts: men with non-metastatic prostate cancer treated with external beam radiation therapy (EBRT) and those on active surveillance (AS) experiencing psychoneurological symptom clusters.

## Subjects and methods

### Subjects

The study was approved by the Institutional Review Board (IRB) of the National Institutes of Health (NIH) (NCT00852111). Study participants were recruited from the NIH Clinical Center between 2009 and 2014. Informed consents were obtained prior to study procedures. Men with non-metastatic prostate cancer, who were scheduled to receive EBRT (*n* = 40), were recruited. Study samples and instruments were collected at two time points: at the start of EBRT (T1) and 3 months after completion of EBRT (T2). The inclusion criteria for the EBRT cohort were: ≥18 years of age, scheduled to receive EBRT with or without concomitant androgen deprivation therapy, and the ability to provide written informed consent. Men with prostate cancer on AS (*n* = 20) were also recruited and seen at the NIH Clinical Center in one outpatient visit. Participants on AS were included if they had clinically localized prostate cancer, had been placed on AS, and did not receive previous or current treatment for prostate cancer. A convenience sample size was selected of all participants with available blood collection and complete clinical and behavioral outcomes.

### Instruments

#### The Functional Assessment Cancer Therapy-Fatigue (FACT-F)

Fatigue levels were assessed using the FACT-F, a validated fatigue assessment tool with a 13-item fatigue scale (Pearson *r* = 0.87, Cronbach alpha = 0.93–0.95) [15, 16] using a Likert scale rated from “not at all = 0” to “very much = 4”. The total scores range from 0 to 52. The lower the score, the greater the intensity and interference of fatigue on daily life.

#### Hamilton Depression Scale (HAM-D)

HAM-D is a 21-item scale to assess the severity of depressive symptoms. This questionnaire is reported to have good internal reliability (Cronbach alpha 0.46-0.97), inter-rater reliability (Pearson *r* = 0.82–0.98), and retest reliability (*r* = 0.81–0.98) [17]. In the clinic, HAM-D scores of 0–7 indicates no depression, 8–13 mild depression, 14–18 moderate depression, 19–22 severe depression, and ≥23 very severe depression [18]. For the purposes of this study, continuous values were used with higher values indicating greater severity [18].

#### Sleep disturbances

The Patient Reported Outcomes Measurement Information System (PROMIS) Sleep Disturbances and Sleep Related Impairment (SD and SRI) is an eight-item questionnaire using a Likert scale rated from “not at all” to “very much” with previously established construct validity and reliability coefficient of 0.90 [19]. Like other PROMIS measures, the PROMIS Sleep Disturbances and Sleep Related Impairment raw scores were converted to *T*-scores to distinguish the responses from the US general population (*T*-score of >50 ± 10 SD). These two questionnaires assess the severity of sleep disturbances and sleep-related impairment [19, 20].

### Revised Piper Fatigue Scale (rPFS) (cognitive/mood fatigue subscale)

The rPFS is a 22-item questionnaire that includes four fatigue dimensions: behavioral/severity (six items), sensory (five items), affective meaning (five items), and cognitive/mood (six items). Only the cognitive/mood fatigue subscale was used in this study, with higher scores indicating greater cognitive fatigue severity. Cronbach’s alpha for rPFS subscales have been reported as 0.89–0.97 [21].

### Blood collection

Peripheral blood was drawn by the NIH Clinical Center staff and collected using ethylenediaminetetraacetic acid (EDTA) tubes. Blood was processed for plasma following standard NINR procedures and plasma was stored at −80 °C until analysis. The primary author ran all the samples together; hence, was blinded to individual subject symptom scores during the experiment (multiple assay).

### EV isolation

EVs were isolated from thawed plasma using ExoQuick^TM^ (SystemBio, Palo Alto, CA) following the manufacturer’s instructions. Each plasma sample was centrifuged twice at 3000*g* × 15 min to deplete the sample of platelets and arrive at platelet poor plasma (PPP). Platelet depleted 250 µl of plasma was incubated with 63 µl of Exoquick^TM^ for 30 min at 4 °C. The sample was centrifuged at 1500*g* × 30 min at 4 °C, the EV-free supernatant was collected, and the EV pellet was resuspended in 250 µl of sterile PBS. Multiplexed bead-based assays were run the same day as the sample preparation. EVs were characterized for size, morphology, and presence of CD markers, as previously described [22].

### Cytokine and heat-shock protein measurements

Concentrations of various inflammatory cytokines and stress-related proteins were measured from total EVs isolated from plasma and were assessed using multiplexed bead-based assays (Luminex). Two panels of markers included IL-1α, IL-1β, IL-2, IL-4, IL-6, IL-7, IL-8, IL-10, IL-12p70, IL-13, IL-15, IL-16, IL-17, IL-18, IL-21, IL-22, IL-33, Calgranulin A (S100A8), Eotaxin (CCL11), granulocyte–macrophage colony-stimulating factor (GM-CSF), growth-regulated alpha (GRO-α or CXCL1), interferon-γ (IFN-γ), interferon-γ-induced protein (IP-10 or CXCL10), interferon-inducible T cell alpha chemoattractant (ITAC or CXCL11), macrophage colony-stimulating factor (M-CSF), monocyte chemoattractant protein-1 (MCP-1 or CCL2), monokine induced by IFN-γ (MIG or CXCL9), macrophage inflammatory protein-1α (MIP-1α or CCL3), MIP-1β (CCL4), MIP-3α (CCL20), regulated on activation normally T cell expressed and secreted (RANTES or CCL5), transforming growth factor-β (TGF-β), and tumor necrosis factor-α (TNF-α), IL-3, IL-6 receptor (R), IL-9, brain-derived neurotrophic factor (BDNF), TNF-related apoptosis-inducing ligand (TRAIL), C-reactive protein (CRP), stromal derived factor (SDF), survivin, and interferon alpha 2 (IFNα2), heat-shock protein (HSP) 27, HSP70, HSP90.

Magnetic beads with distinct spectral signatures were coupled with protein-specific capture antibodies to allow measurement of many different proteins in a single low-volume sample. Catalog numbers and manufacturers for capture and detection antibodies are provided in Supplementary Table 1. Standards (unlysed or lysed with 0.1% Triton X-100), intact and lysed EVs, and EV-free supernatants were diluted in assay buffer and then incubated with bead mixes overnight at 4 °C. The next day, plates were washed two times and incubated with polyclonal antibodies for 1 h. After two washes, plates were incubated with streptavidin-phycoerythrin (16 μg/ml) in PBS (Thermo Fisher). After final washes, beads were resuspended in PBS and cytokines were read on a Luminex 200 System and analyzed with Bioplex Manager software (BioRad). The protocols were previously tested and published [14, 22].

Final concentrations of soluble analytes measured in EV-free supernatants were adjusted to account for dilution by ExoQuick ^TM^ reagent. Surface EV protein was reported by measurement on intact EVs, and internal EV protein was calculated from EV lysed concentrations (lysed minus surface equals internal). Total EV-associated cytokines (surface plus internal) were used for analysis in this study. Lower limits of detection (LLOD) and standard curves were determined using 5P logistic regression. Data on LLOD for each analyte and descriptive statistics for EV concentrations for EBRT cohort at T1 and T2 has been previously published [22]. Data on AS group’s EV concentrations (mean and standard deviation) are included in Supplementary Material Table 2.

### Statistical analyses

**Fig. 2:**
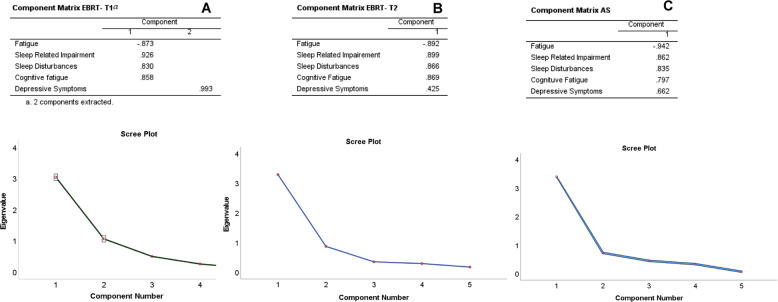
Principal component analysis results. **A** Two components were extracted, component 1 had an initial eigenvalue of 3.05 with 60.9% of variance and component 2 had an initial eigenvalue of 1.01 with 21% of variance demonstrated by scree plot. Component 2 did not meet clinical definition for symptom cluster [25]; thus, only component 1 is included. Assumptions for PCA met: KMO for sampling adequacy (0.676) and Bartlett’s test Chi square for suitability for data reduction (92.8, *p* < 0.0001). **B** One component was identified for EBRT-T2 cluster with an initial eigenvalue of 3.3 and 65.8% of total variance. The corresponding scree plot illustrates the component. Assumptions met included KMO for sampling adequacy (0.82) and Bartlett’s test Chi square for suitability for data reduction (102.9, *p* < 0.0001). **C** One component was also identified for AS cluster with initial eigenvalue of 3.4 and 68% of variance. Corresponding scree plot illustrates the component. Assumptions for testing included KMO for sampling adequacy (0.701) and Bartlett’s test Chi square for suitability for data reduction (58.9, *p* < 0.0001).

Descriptive statistics characterized the demographic, clinical, and symptom attributes of the study participants. Data were evaluated for Gaussian distribution by calculating skewness and kurtosis statistics (Supplementary Material Table 3). Two-sided Mann–Whitney *U* test was used to evaluate group differences between EBRT and AS cohorts for non-normally distributed variables and two-sided independent *T-*test was used to evaluate group differences for normally distributed variables. Two-sided paired *T-*test was used to evaluate differences between EBRT-T1 and EBRT-T2 for normally distributed variables and two-sided Wilcoxon signed-rank test was used to evaluate differences between EBRT-T1 and EBRT-T2 for non-normally distributed variables. Pearson chi square test was used for categorical variables. Due to non-normal distributions of the variables, cytokine concentrations were logarithm base ten transformed (Supplementary Table 4). The following symptoms: fatigue, sleep-related impairment, sleep-related disturbances, depression, and cognitive fatigue for each cohort were entered into SPSS to calculate symptoms clusters using principal components analysis (PCA). The symptom clusters identified by PCA were then correlated with the concentrations of logarithm base ten transformed concentrations of the 45 biomarkers using Pearson correlation tests. Assumptions met for PCA analysis are presented in Fig. 2 description. All statistically significant values are indicated by *p* < 0.05. IBM SPSS Grad pack 24.0 was used to conduct all statistical analyses. JMP™ Statistical Discovery Software (SAS Headquarters, Cary, NC) was used for hierarchical clustering using the Ward distance metric for both the EV and soluble log ten concentrations. Due to small sample size and explorative nature of the analysis, multiple comparison corrections were not performed. Data are presented with mean as measure of center values and standard deviation as measure of estimate of variation.

### Missing data

Five patients in the EBRT group did not complete the sleep disturbances and sleep-related impairment forms at T1 and one participant did not complete the rPFS mood/cognitive subscale questionnaire at T2. The final data were calculated on the remaining 35 individuals for sleep disturbances and sleep-related impairment questionnaires and 39 individuals for the rPFS mood/cognitive subscale. Two responses (1, 2) were marked on the SRI form by one of the participants. Following the PROMIS SRI scoring manual, a data specialist picked one of the two responses by flipping a coin and the selected response was entered into the dataset.

## Results

Clinical and demographic attributes of participants in each group are presented in Table 1. AS men had significantly lower T-stage levels, lower Gleason scores and higher levels of red blood cell (RBC) counts than EBRT men (*p* < 0.05). RBC, hemoglobin, and prostate-specific antigen (PSA) were significantly lower at EBRT-T2 compared to EBRT-T1 (*p* < 0.001).

**Table 1:** Clinical, demographic, and symptoms characteristics of study participants.

	EBRT-T1 (*n* = 40)	EBRT-T2 (*n* = 40)	AS (*n* = 20)	*p* (EBRT-T1 vs. AS)	*p* (EBRT-T1 vs. EBRT-T2)
Age (years)	66.9 (7.3)		65.5 (9.11)	0.531	
BMI	29.7 (4.5)		28.18 (4.9)	0.214	
Radiation dose		7572 (276.2)			
*Race/Ethnicity*				0.465	
White	67.5%		85.0%		
Black/African American	25.0%		10.0%		
Asian	6.0%		5.0%		
Hispanic	2.5%				
*T -stage*				0.008*	
T0			20.0%		
T1c	32.5%		60.0%		
T2a	40.0%		20.0%		
T2b	5.0%				
T2c	5.0%				
T3a	10.0%				
T3b	7.5%				
*Gleason Scores*				0.000*	
3 + 3 = 6	7.5%		80.0%		
3 + 4 = 7	32.5%		15.0%		
4 + 3 = 7	12.5%		5.0%		
3 + 5 = 8	2.5%				
4 + 4 = 8	12.0%				
5 + 4 = 9	5.0%				
4 + 5 = 9	10.0%				
ADT	80.0%				
Surgery	10.0%				
Hb (g/dL)	13.95 (1)	12.9 (1.1)	14.49 (0.92)	0.06	0.000*
RBC (μL)	4.6 (0.45)	4.26 (0.45)	4.9 (0.46)	0.017*	0.000*
PSA (ng/mL)	7.85 (16.8)	0.31 (0.79)	5.76 (6.05)	0.510	0.000*
Fatigue (FACT-F)	44 (6.6)	41.95 (9.6)	44.5 (6.7)		
Depression (HAM-D)	0.85 (1.3)	0.95 (1.3)	1.1 (2.4)		
Sleep Disturbances (PROMIS SD)	47 (10.3)	45 (10)	46.5 (9.2)		
Sleep Related Impairment (PROMIS SRI)	42.5 (10)	43 (10)	44.6 (10.2)		
Cognitive Fatigue (rPFS)	1.95 (1.7)	2.6 (2.3)	1.7 (1.6)		

Two-sided Mann–Whitney *U* test was used to evaluate group differences between EBRT and AS cohorts for PSA. Two-sided Independent *T-*test was used to evaluate group differences between EBRT and AS cohorts for normally distributed variables (age, BMI, RBC, and HB). Two-sided Wilcoxon signed-rank test was used to evaluate differences between EBRT-T1 and EBRT-T2 for PSA. Two-sided paired *T-*test was used to evaluate differences between EBRT-T1 and EBRT-T2 for normally distributed variables (RBC and HB).

*BMI* body mass index, *ADT* androgen deprivation therapy, *HB* hemoglobin, *RBC* red blood cells, *PSA* prostate-specific antigen, *FACT-F* the Functional Assessment Cancer Therapy-Fatigue, *HAM-D* Hamilton Depression Rating Scale, *PROMIS* patient-reported outcome measurement information system, *SD* sleep disturbances, *SRI* sleep-related impairment, *rPFS* revised Piper Fatigue Scale, *EBRT* external beam radiation therapy, *AS* active surveillance. *Statistically significant.

### Symptom clusters

Principal component analysis was performed on raw symptom scores for each cohort at each time point. Assumptions for sampling adequacy and suitability for data reduction were demonstrated with Kaiser–Meyer–Olkin (KMO) measures and Bartlett’s test [23, 24]. The psychoneurological symptom cluster in EBRT-T1 included all symptoms (CRF, sleep disturbance/sleep impairment, cognitive fatigue). Depressive symptoms (assessed by HAM-D) formed a smaller independent component (Fig. 2) and did not meet clinical criteria for symptom clusters previously defined to include three or more concurrent symptoms [25]. Therefore, it was not counted as part of the cluster. Symptom clusters for EBRT-T2 and AS included all four symptoms. Component matrix and scree plots for each symptom cluster are presented in Fig. 2A–C.

### EV- associated and soluble markers

**Fig. 3:**
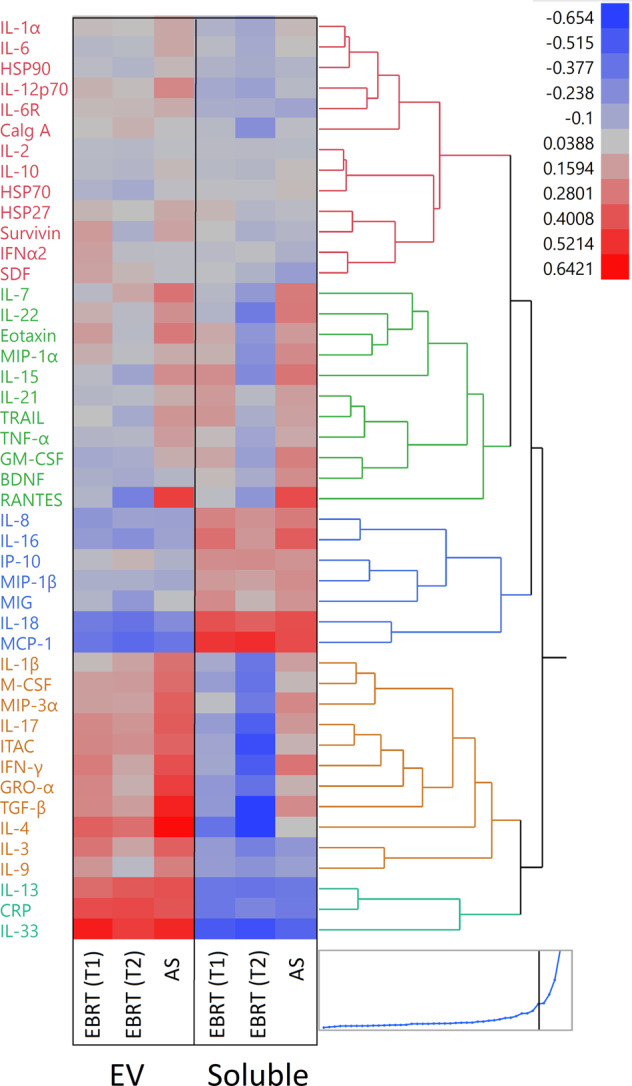
Hierarchical cluster of average cytokines, chemokines, and heat-shock protein expression in logarithm base ten from multiplexed bead-based arrays. Clustering shows the relative differences in concentrations of the markers between EV and soluble fractions. The five different color-coded dendrograms shows clusters of biomarkers with similar expression values across the EV and soluble fractions. EBRT external beam radiation therapy, T1 at the start of radiation, T2 3 months post radiation, AS active surveillance.

Hierarchical clustering analysis was used to visualize average marker concentrations in soluble and EV-associated fractions for all three groups (EBRT-T1, EBRT-T2, and AS) (Fig. 3). The hierarchical clustering dendrogram was colored at the level of five separate dendrogram breaks to indicate those markers that were expressed similarly across participant groups. As shown in Fig. 3, the first break in the dendrogram (labeled as the teal and orange dendrograms) represent markers that are more expressed in EVs vs. soluble in the EBRT-T1&2 groups. These markers include IL-1b, M-CSF, MIP-3α, IL-17, ITAC, GRO-α, TGF-β, IL-4, IL-3, IL-9, IL-13, CRP, and IL-33.

### Correlation of marker concentrations with psychoneurological symptom clusters

We investigated the correlations of the concentrations of EV-associated and soluble markers with the identified symptom clusters. Each composite symptom score was correlated with the concentrations of the 45 markers in EV-associated and soluble fractions (Table 2). High component scores are indicative of worse symptoms and positive relationships signify that higher biomarker concentrations are associated with worse symptom scores. Negative correlations indicate that higher cytokine/protein concentrations are associated with lower symptom severity.

**Table 2:** Bivariate correlations between EBRT-T1, EBRT-T2, and AS symptom clusters and soluble and EV-associated immune markers.

	Pearson *r*	*P* value
*Soluble cytokine correlations with EBRT-T1 cluster*		
RANTES	0.380*	0.024
IL-21	−0.479**	0.004
IL-33	−0.346*	0.042
*EV-associated cytokine correlations with EBRT-T1 cluster*		
IL-1α	−0.375*	0.026
IL-15	−0.465**	0.005
IL-21	−0.345*	0.042
GM-CSF	−0.359*	0.034
M-CSF	−0.335*	0.049
RANTES	0.421*	0.012
*Soluble cytokine correlations with EBRT-T2 cluster*		
IFNα2	0.413**	0.009
IL-9	0.447**	0.004
IL-17	0.371*	0.02
*Soluble cytokine correlations with AS cluster*		
HSP90	−0.446*	0.049
Survivin	0.474*	0.035
IL-1b	−0.451*	0.046
*EV-associated cytokine correlations with AS group*		
HSP70	−0.478*	0.033
MIG	−0.548*	0.012

*EBRT* external beam radiation therapy, *AS* active surveillance, *T1* at the start of radiation.

*p < 0.05, **p < 0.01.

For the EBRT-T1 psychoneurological symptom cluster, only RANTES had significant positive correlation, indicating worsening symptoms. No significant correlations were found between EV-associated markers and EBRT-T2 psychoneurological symptom cluster. However, there was positive correlation between soluble IFNα2, IL-9, and IL-17 and this symptom cluster. AS psychoneurological symptom cluster correlated positively only with soluble survivin.

## Discussion

We investigated the associations of EV and soluble immune markers with psychoneurological symptom clusters in men with prostate cancer. We found that: (i) symptoms cluster profiles differed at various cancer treatment trajectories (among AS cohort, before EBRT, and at 3 months post EBRT); (ii) EV-associated and soluble markers correlated with individual symptom clusters (composite scores).

Upregulation of chemokines and cytokines have been previously reported to be associated with worsening fatigue [26], depressive symptoms [27], and cognitive disturbances [28] in patients with cancer. We hypothesized that the proinflammatory immune markers reflect worsening psychoneurological state in patients with prostate cancer.

Our study revealed that at the start of EBRT, soluble and EV-associated RANTES increased with worsening symptoms within EBRT-T1 symptom cluster. RANTES is a member of the chemokine family predominantly produced by CD8 T cells and associated with various inflammatory disorders [29]. An earlier study reported that elevated expression of RANTES correlated with severe general fatigue in patients with lung cancer after tyrosine kinase inhibitor treatment [9]. Furthermore, RANTES was reported to be elevated in men and women with different levels of depression severity [30].

At T2, we found that several soluble markers (IFNα2, IL-9, and IL-17) were associated with worse symptoms within EBRT-T2 psychoneurological symptom cluster. All three cytokines share a common signaling pathway through the NFκB complex: IFNα2 increases NFκB activity through IFN-induced genes [31], IL-17 leads to the activation of NFκB [32], and NFkB has been implicated in the expression of IL-9 [33]. Increase in NFκB transcription factors has been previously linked to persistent fatigue in patients with breast cancer [34], and linked to sleep disturbances in healthy adults [35] as well as in patients with long-standing depressive symptoms [36]. This suggests that an increase in proinflammatory cytokines may be associated with the neuropsychological symptom cluster 3 months after treatment completion.

Several publications reported on the association of individual cytokines roles in various psychoneurological symptoms. It was established that cytokine therapy with IFNα is associated with the development of depressive symptoms in patients with malignant melanoma [37]. Furthermore, it was found IFNα therapy for malignant melanoma led to symptoms of fatigue, loss of concentration, gastrointestinal symptoms, and tension/irritability in addition to depressed mood [37]. IFNα treatment in hepatitis C correlated with fatigue, depressed mood, and anxiety ratings [38]. In agreement of these studies, we found that at EBRT-T2, depressive symptoms interrelated with remaining psychoneurological symptoms in PCA and significantly correlated with IFNα2.

Another cytokine that had positive correlation with EBRT-T2 symptom cluster was IL-9. It is a pleiotropic cytokine known to have a dual immunomodulating effect, which largely depends on what subset of Th cells are responsible for its production [39]. Our finding is consistent with a previously reported correlation of IL-9 concentration with worsening fatigue in men with prostate cancer after radiation [26] and chronic fatigue in patients with non-Hodgkin’s lymphoma [40].

Also, we found that an increase in IL-17 levels correlated with worsening psychoneurological symptoms 3 months after completing radiation therapy. IL-17 is part of six structurally related IL-17 family cytokines known to induce a proinflammatory response in cancer and autoimmune disease [41]. While our study is the first to correlate IL-17 to a psychoneurological symptom cluster, previous reports have shown increased levels of IL-17 to be associated with other psychoneurological symptoms such as major depressive disorder [42] and anxiety in rheumatoid arthritis [43].

In comparison to the EBRT cohorts, in AS men, bivariate correlation analysis showed positive correlation between high concentrations of survivin and increased intensity of psychoneurological symptoms. Survivin is a member of an inhibitor of apoptosis family distinctly expressed in tumors [44]. We previously showed soluble survivin to be associated with cancer-related fatigue [22] in men with prostate cancer 3 months after radiation therapy. While published data support evidence for survivin leading to poor outcomes with cancer, to our knowledge, the present study is the first to examine survivin in relation to psychoneurological cancer-related symptoms.

In summary, we reported that both EBRT-T2 and AS clusters have the same set of symptoms closely interrelated within the cluster, but different immune biomarkers differentially correlated with each of these clusters. This distinction can be attributed to the differences in the clinical trajectories and clinical outcomes. We identified specific symptom clusters for each study group and distinct cytokines that were associated with each cluster. RANTES was the only chemokine that was significant in both EV-associated and soluble forms. In our previous report on men with prostate cancer before and after EBRT, we found more EV-associated cytokines were statistically significant correlated with worsening fatigue compared to soluble ones when looking at change in total concentration of cytokines over time [22]. Comparison of EV-associated and soluble cytokines changes can be informative in discerning inflammatory pathways related to psychoneurological symptom clusters.

Our exploratory study has several limitations. First, the sample size was relatively small, thus preventing multiple testing correction. Second, blood collections were performed at different times of the day, which may introduce diurnal variation [45]. A third limitation of this study is that samples were collected from 2009 to 2014 and prolonged storage may have led to slow protein degradation. A study that evaluated stability of cytokines in plasma over 4 years concluded that IL-13, IL-15, IL-17, and CXCL8 showed degradation within one year, whereas IL-2, IL-4, IL-12, and IL-18 were stable up to 3 years. Other cytokines (Il-1a, IL-1B, IL-5, IL-6, and IL-10) had degradation up to 50% within 2–3 years of storage [46]. There are no data in the literature regarding the stability of EVs over long-term storage. In the current study, both control and experimental samples were stored in the same way and were processed for EV extractions and multiplex assays on the same day after defrosting samples to avoid repeated thaw freeze cycles. Lastly, we did not study which cell types the EVs in this study are most likely originating from and/or half-life of cytokines that are free vs. EV-associated. There are no published data regarding the possible difference between half-life of free and EV-associated cytokines. In general, cytokines are relatively stable compounds [46]. A recent study examining stability of EV proteins, RNA, and other EV markers at RT, 4 °C, and −70 °C found that CD63 and hsp70found that faster degradation of CD63 and HSP70 was reached when incubated at RT and 4 °C compared to 10 days at −70 °C [47]. We stored our samples at −80 °C. Because most of clinical research takes years for initial data collection, future studies with longer storage (5–10 years) should investigate the effect of degradation and half-life of cytokines in soluble form and EV associated. In relation to cell-specific origin of EVs, it is plausible that some EV-associated cytokines are originating from prostate cancer cells, muscle cells, vascular origin, or neuronal origin. Numerous studies have recently shown that EVs may cross the blood–brain barrier and neuronal EV-associated biomarkers have been detected in circulating plasma using an immunoaffinity assay (e.g. EV + CD171) [11, 48].

Nevertheless, these findings are of important clinical relevance as they suggest that cancer and cancer treatments may drive co-occurrence of symptoms resulting in specific symptom clusters. The cytokines that positively correlated with worsening symptoms have known pro- inflammatory effects, which supports our observation that inflammation is associated with a worsening psychoneurological state in patients with prostate cancer. This observation supports the notion that worsening symptoms may be affected by the inflammatory processes and changes in these processes (worsening or improvement of inflammation) have a consequential effect on the symptom clusters. The knowledge that EV-associated cytokines significantly correlate with worsening symptoms may unravel distinct pathways. Future studies will reveal the roles of specific EVs, mainly, neuronally derived ones and their association of cytokines to separate the impact of central vs. peripheral inflammation.

## Supplementary information

Supplementary Material

## Data Availability

The data that support the findings of this study are available on reasonable request to the corresponding author.
