# Supplementary material for: Extracellular vesicle associated and soluble immune marker profiles of psychoneurological symptom clusters in men with prostate cancer: an exploratory study
Source: Transl Psychiatry. 2021 Aug 24;11:440. doi: 10.1038/s41398-021-01554-w (PMC8385103; doi:10.1038/s41398-021-01554-w)
Supplement: Supplementary file 1 — Supplementary Material [file 41398_2021_1554_MOESM1_ESM.docx]

**Supplemental Table 1.** Vendor and catalog numbers for 45 capture and detection antibodies used in multiplexed bead assay.

| **Immune Marker** | **Capture Antibody** | **Detection Antibody** |
| --- | --- | --- |
| BDNF | R&D MAB848 | R&D BAM648 |
| CRP | R&D MAB17071 | R&D BAM17072 |
| HSP27 | RayBiotech 119-11560 | R&D AF15801 |
| HSP70 | Origene TA60030 | R&D AF1663 |
| HSP90 | RayBiotech 119-13332 | AssayPro 20290-05021 |
| IFNα2 | Biolegend 537102 | Biolegend 537204 |
| IL-3 | Biolegend 500502 | Biolegend 500604 |
| IL-9 | Biolegend 507602 | Biolegend 507704 |
| IL-6R | R&D MAB227 | R&D BAF227 |
| SDF-1-a | R&D MAB350 | R&D BAF310 |
| Survivin | R&D MAB886 | R&D AF6471 |
| TRAIL | R&D MAB3751 | R&D BAF375 |
| IL-1α | R&D MAB200 | R&D BAF200 |
| IL-1β | R&D MAB601 | R&D BAF201 |
| IL-2 | R&D MAB602 | R&D BAF202 |
| IL-4 | Biolegend 500707 | Biolegend 500804 |
| IL-6 | R&D MAB206 | R&D BAF206 |
| IL-7 | R&D MAB207 | R&D BAF207 |
| IL-8 | R&D MAB208 | R&D BAF208 |
| IL-10 | R&D MAB2172 | R&D BAF217 |
| IL-12 p70 | BD 555065 | R&D BAF219 |
| IL-13 | R&D MAB213 | R&D BAF213 |
| IL-15 | R&D MAB647 | R&D BAM247 |
| IL-16 | R&D MAB316 | R&D BAF316 |
| IL-17 | R&D MAB317 | R&D BAF317 |
| IL-18 | R&D D044-3 | R&D D045-6 |
| IL-21 | Invitrogen 14-7219-82 | R&D AF15001 |
| IL-22 | R&D MAB7822 | R&D BAM7821 |
| IL-33 | R&D MAB3625 | R&D BAF3625 |
| Calgranulin A | R&D MAB4570 | R&D BAF4570 |
| Eotaxin | R&D MAB320 | R&D BAF320 |
| GM-CSF | R&D MAB615 | R&D BAM215 |
| Gro-α | R&D MAB275 | R&D BAF275 |
| IFN- g | R&D MAB285 | R&D BAF285 |
| IP-10 | R&D MAB266 | R&D BAF266 |
| ITAC | R&D MAB672 | R&D BAF672 |
| M-CSF | R&D MAB616 | R&D BAF216 |
| MCP-1 | R&D MAB679 | R&D BAF279 |
| MIG | R&D MAB392 | R&D BAF392 |
| MIP-1α | R&D MAB670 | R&D BAF270 |
| MIP-1β | R&D MAB271 | R&D BAF271 |
| MIP3-α | R&D MAB360 | R&D BAF360 |
| TGF-β | R&D MAB240 | R&D BAF240 |
| TNF-α | R&D MAB610 | R&D BAF210 |
| RANTES | R&D MAB678 | R&D BAF278 |

**Supplemental Table 2.** Descriptive statistics of EV-associated and soluble concentrations (pg/ml) of immune markers measured by Luminex in men with prostate cancer on active surveillance.

| **EV - Associated** | | **Soluble** | |
| --- | --- | --- | --- |
| Immune Marker | Mean (SD) | Immune Marker | Mean (SD) |
| BDNF | 564.31 (200.60) | BDNF | 1162.60 (1010.67) |
| CRP | 608832.19 (247593.73) | CRP | 185507.71 (262488.82) |
| HSP27 | 1305.05 (750.17) | HSP27 | 833.64 (310.89) |
| HSP70 | 29.01 (16.82) | HSP70 | 33.02 (6.15) |
| HSP90 | 111.69 (76.44) | HSP90 | 78.72 (17.05) |
| IFNα2 | 285.82 (1150.02) | IFNα2 | 34.76 (59.12) |
| IL-3 | 77.78 (83.10) | IL-3 | 31.93 (33.13) |
| IL-9 | 19.08 (42.82) | IL-9 | 1.95 (2.86) |
| IL-6R | 1147.21 (386.85) | IL-6R | 670.58 (195.96) |
| SDF-1 | 2324.86 (1190.88) | SDF-1 | 2031.07 (1251.51) |
| Survivin | 14.18 (16.00) | Survivin | 8.16 (7.89) |
| TRAIL | 404.46 (551.40) | TRAIL | 254.25 (145.72) |
| IL-1α | 2.31 (6.14) | IL-1α | 1.00 (1.35) |
| IL-1β | 10.16 (8.92) | IL-1β | 6.33 (3.13) |
| IL-2 | 1.31 (2.95) | IL-2 | 0.73 (0.03) |
| IL-4 | 103.10 (221.50) | IL-4 | 14.22 (10.87) |
| IL-6 | 1.59 (2.55) | IL-6 | 0.86 (0.86) |
| IL-7 | 46.17 (58.69) | IL-7 | 28.59 (16.40) |
| IL-8 | 2.77 (6.26) | IL-8 | 5.74 (3.51) |
| IL-10 | 5.64 (21.35) | IL-10 | 1.43 (1.75) |
| IL-12 p70 | 6.14 (14.31) | IL-12 *p*70 | 1.06 (1.21) |
| IL-13 | 116.55 (405.93) | IL-13 | 4.62 (2.48) |
| IL-15 | 32.94 (55.99) | IL-15 | 16.74 (5.98) |
| IL-16 | 269.29 (148.25) | IL-16 | 803.42 (384.51) |
| IL-17 | 36.83 (56.07) | IL-17 | 25.00 (23.79) |
| IL-18 | 2.76 (1.71) | IL-18 | 16.22 (7.63) |
| IL-21 | 817.91 (1195.61) | IL-21 | 678.25 (355.46) |
| IL-22 | 67.46 (64.10) | IL-22 | 67.30 (38.63) |
| IL-33 | 397.89 (930.51) | IL-33 | 33.44 (71.58) |
| Calgranulin A | 1.62 (0.00) | Calgranulin A | 7.76 (23.06) |
| Eotaxin | 1146.99 (1093.72) | Eotaxin | 857.60 (321.78) |
| GM-CSF | 15.16 (14.35) | GM-CSF | 17.34 (6.70) |
| Gro-α | 283.41 (404.14) | Gro-α | 114.10 (122.76) |
| IFN- g | 347.24 (617.17) | IFN-g | 155.01 (83.50) |
| IP-10 | 1583.52 (1590.38) | IP-10 | 2429.47 (1863.19) |
| ITAC | 1568.30 (1828.29) | ITAC | 721.35 (382.07) |
| M-CSF | 22.40 (41.40) | M-CSF | 9.05 (6.92) |
| MCP-1 | 11.10 (10.94) | MCP-1 | 65.73 (32.66) |
| MIG | 1313.24 (693.88) | MIG | 2000.83 (1308.16) |
| MIP-1α | 12.70 (15.00) | MIP-1α | 12.25 (6.27) |
| MIP-1β | 11.80 (8.52) | MIP-1β | 23.89 (12.99) |
| MIP3α | 449.30 (433.38) | MIP3α | 223.22 (106.00) |
| TGF-β | 435.04 (804.91) | TGF-β | 107.68 (76.78) |
| TNF-α | 5.18 (6.82) | TNF-α | 3.11 (1.24) |
| RANTES | 23552.33 (19187.04) | RANTES | 25966.52 (18745.65) |

**Supplemental Table 3.** Normality statistics for demographic, clinical, and biomarker variables.

| EBRT Group | | | | | | | | | |
| --- | --- | --- | --- | --- | --- | --- | --- | --- | --- |
|  | N | Mean | Std. Deviation | Skewness |  | Kurtosis |  | Skewness/ | Kurtosis/ |
|  | Statistic | Statistic | Statistic | Statistic | Std. Error | Statistic | Std. Error | SE(Skew) | SE(Kurtosis) |
| EBRT | | | | | | | | | |
| Age* | 40.00 | 66.93 | 7.33 | -0.21 | 0.37 | -0.53 | 0.73 | -0.57 | -0.72 |
| BMI* | 40.00 | 29.80 | 4.52 | 0.79 | 0.37 | 1.02 | 0.73 | 2.11 | 1.39 |
| Total dose*** | 40.00 | 7571.50 | 276.23 | -1.75 | 0.37 | 4.86 | 0.73 | -4.69 | 6.64 |
| RBC T1* | 40.00 | 4.61 | 0.45 | 0.46 | 0.37 | 0.78 | 0.73 | 1.24 | 1.07 |
| PSA T2** | 39.00 | 0.31 | 0.79 | 4.29 | 0.38 | 20.69 | 0.74 | 11.34 | 27.92 |
| PSA T1** | 40.00 | 7.85 | 16.82 | 5.21 | 0.37 | 30.07 | 0.73 | 13.93 | 41.04 |
| HB T1* | 40.00 | 13.95 | 1.06 | 0.32 | 0.37 | -0.37 | 0.73 | 0.85 | -0.51 |
| HB T2* | 40.00 | 13.00 | 1.13 | 0.23 | 0.37 | -0.47 | 0.73 | 0.61 | -0.64 |
| RBC T2* | 40.00 | 4.27 | 0.46 | 0.55 | 0.37 | 1.05 | 0.73 | 1.46 | 1.44 |
| Active Surveillance | | | | | | | | | |
| Age* | 20.00 | 65.55 | 9.12 | 0.29 | 0.51 | -0.38 | 0.99 | 0.57 | -0.38 |
| BMI* | 20.00 | 28.19 | 4.99 | 0.10 | 0.51 | -0.30 | 0.99 | 0.20 | -0.30 |
| RBC T1* | 20.00 | 4.92 | 0.46 | 1.10 | 0.51 | 1.73 | 0.99 | 2.16 | 1.74 |
| PSA T1** | 20.00 | 5.77 | 6.05 | 3.06 | 0.51 | 10.80 | 0.99 | 5.97 | 10.89 |
| HB T1* | 20.00 | 14.49 | 0.92 | 0.00 | 0.51 | 0.94 | 0.99 | -0.01 | 0.95 |

**Abbreviations:** EBRT = External Beam Radiation **Therapy** BMI = Body Mass Index, RBC = Red Blood Cell, PSA = Prostate Specific Antigen, Hb = Hemoglobin, T1 = Start of EBRT, T2 = 6-months post-EBRT. ***only EBRT T1 received treatment.

Data was evaluated for normality distribution by generating the ratios of the statistic/Std.Err for both the skewness and kurtosis. If these ratios were outside the bounds of (-2,2), then the distribution was considered non-normal. * normal distribution, ** non-normal distribution.

**Supplemental Table 4.** Normality statistics for EV-associated and soluble concentrations (pg/ml) of immune markers.

|  | N Statistic | Skewness Statistic | Std. Error | kurtosis Statistic | Std. Error | Skewness statistics/SE | Kurtosis statistics/SE |
| --- | --- | --- | --- | --- | --- | --- | --- |
| EBRT T1 Group | | | | | | | |
| EV-associated | | | | | | | |
| BDNF | 40.00 | 0.16 | 0.37 | -0.45 | 0.73 | 0.42 | -0.62 |
| CRP | 40.00 | -0.25 | 0.37 | -0.55 | 0.73 | -0.68 | -0.75 |
| HSP27 | 40.00 | 1.58 | 0.37 | 3.22 | 0.73 | 4.22 | 4.39 |
| HSP70 | 40.00 | 1.95 | 0.37 | 5.26 | 0.73 | 5.21 | 7.17 |
| HSP90 | 40.00 | 2.64 | 0.37 | 7.93 | 0.73 | 7.07 | 10.82 |
| IFNα2 | 40.00 | 4.02 | 0.37 | 18.37 | 0.73 | 10.75 | 25.08 |
| IL-3 | 40.00 | 3.11 | 0.37 | 11.58 | 0.73 | 8.31 | 15.81 |
| IL-9 | 40.00 | 5.18 | 0.37 | 29.26 | 0.73 | 13.85 | 39.95 |
| IL-6R | 40.00 | 0.59 | 0.37 | 0.17 | 0.73 | 1.59 | 0.23 |
| SDF | 40.00 | 0.05 | 0.37 | 0.00 | 0.73 | 0.13 | 0.00 |
| Survivin | 40.00 | 4.64 | 0.37 | 24.24 | 0.73 | 12.42 | 33.08 |
| TRAIL | 40.00 | 2.02 | 0.37 | 4.40 | 0.73 | 5.41 | 6.01 |
| IL-1α | 40.00 | 3.57 | 0.37 | 15.88 | 0.73 | 9.55 | 21.68 |
| IL-1β | 40.00 | 0.88 | 0.37 | -0.38 | 0.73 | 2.35 | -0.51 |
| IL-2 | 40.00 | 5.96 | 0.37 | 36.43 | 0.73 | 15.94 | 49.72 |
| IL-4 | 40.00 | 1.16 | 0.37 | -0.22 | 0.73 | 3.11 | -0.31 |
| IL-6 | 40.00 | 0.97 | 0.37 | 0.62 | 0.73 | 2.60 | 0.84 |
| IL-7 | 40.00 | 3.02 | 0.37 | 11.15 | 0.73 | 8.08 | 15.22 |
| IL-8 | 40.00 | 4.92 | 0.37 | 27.65 | 0.73 | 13.15 | 37.74 |
| IL-10 | 40.00 | 4.06 | 0.37 | 16.46 | 0.73 | 10.86 | 22.47 |
| IL-12p70 | 40.00 | 3.27 | 0.37 | 11.45 | 0.73 | 8.74 | 15.63 |
| IL-13 | 40.00 | 6.32 | 0.37 | 40.00 | 0.73 | 16.92 | 54.59 |
| IL-15 | 40.00 | 1.53 | 0.37 | 0.89 | 0.73 | 4.09 | 1.22 |
| IL-16 | 40.00 | 0.50 | 0.37 | -0.11 | 0.73 | 1.35 | -0.15 |
| IL-17 | 40.00 | 1.61 | 0.37 | 1.70 | 0.73 | 4.30 | 2.32 |
| IL-18 | 40.00 | 2.17 | 0.37 | 6.13 | 0.73 | 5.81 | 8.36 |
| IL-21 | 40.00 | 3.64 | 0.37 | 17.88 | 0.73 | 9.73 | 24.40 |
| IL-22 | 40.00 | 1.42 | 0.37 | 1.25 | 0.73 | 3.79 | 1.70 |
| IL-33 | 40.00 | 3.03 | 0.37 | 8.79 | 0.73 | 8.10 | 12.00 |
| Calgranulin | 40.00 | 6.32 | 0.37 | 40.00 | 0.73 | 16.92 | 54.60 |
| Eotaxin | 40.00 | 1.28 | 0.37 | 2.92 | 0.73 | 3.41 | 3.99 |
| GM-CSF | 40.00 | 1.09 | 0.37 | 0.87 | 0.73 | 2.92 | 1.18 |
| Gro-α | 40.00 | 0.60 | 0.37 | -1.13 | 0.73 | 1.60 | -1.55 |
| IFN-γ | 40.00 | 4.46 | 0.37 | 24.17 | 0.73 | 11.94 | 33.00 |
| IP-10 | 40.00 | 2.16 | 0.37 | 4.83 | 0.73 | 5.78 | 6.59 |
| ITAC | 40.00 | -0.03 | 0.37 | -1.57 | 0.73 | -0.08 | -2.14 |
| MCSF | 40.00 | 1.42 | 0.37 | 3.21 | 0.73 | 3.80 | 4.38 |
| MCP-1 | 40.00 | 4.07 | 0.37 | 20.51 | 0.73 | 10.88 | 28.00 |
| MIG | 40.00 | 4.95 | 0.37 | 27.66 | 0.73 | 13.25 | 37.75 |
| MIP-1α | 40.00 | 2.67 | 0.37 | 10.77 | 0.73 | 7.13 | 14.70 |
| MIP-1β | 40.00 | 6.03 | 0.37 | 37.35 | 0.73 | 16.13 | 50.99 |
| MIP-3α | 40.00 | 2.23 | 0.37 | 7.01 | 0.73 | 5.95 | 9.57 |
| TGF-β | 40.00 | 2.14 | 0.37 | 4.47 | 0.73 | 5.73 | 6.11 |
| TNF-α | 40.00 | 1.68 | 0.37 | 3.60 | 0.73 | 4.50 | 4.91 |
| RANTES | 40.00 | 1.46 | 0.37 | 1.13 | 0.73 | 3.91 | 1.54 |
| Soluble | | | | | | | |
| BDNF | 40.00 | 2.29 | 0.37 | 5.10 | 0.73 | 6.13 | 6.96 |
| CRP | 40.00 | 2.30 | 0.37 | 5.05 | 0.73 | 6.17 | 6.90 |
| HSP27 | 40.00 | 2.14 | 0.37 | 5.65 | 0.73 | 5.73 | 7.71 |
| HSP70 | 40.00 | 0.58 | 0.37 | -0.79 | 0.73 | 1.55 | -1.07 |
| HSP90 | 40.00 | 1.03 | 0.37 | 0.65 | 0.73 | 2.75 | 0.89 |
| IFNα-2 | 40.00 | 2.83 | 0.37 | 7.18 | 0.73 | 7.58 | 9.80 |
| IL-3 | 40.00 | 0.87 | 0.37 | -0.09 | 0.73 | 2.32 | -0.12 |
| IL-9 | 40.00 | 1.87 | 0.37 | 2.39 | 0.73 | 5.01 | 3.26 |
| IL-6R | 40.00 | 1.18 | 0.37 | 0.48 | 0.73 | 3.16 | 0.66 |
| SDF | 40.00 | -0.44 | 0.37 | -0.96 | 0.73 | -1.18 | -1.30 |
| SURVIVIN | 40.00 | 0.72 | 0.37 | 0.18 | 0.73 | 1.94 | 0.24 |
| TRAIL | 40.00 | 1.29 | 0.37 | 2.19 | 0.73 | 3.46 | 2.98 |
| IL-1α | 40.00 | 2.06 | 0.37 | 3.57 | 0.73 | 5.52 | 4.87 |
| IL-1β | 40.00 | -0.22 | 0.37 | -1.43 | 0.73 | -0.58 | -1.95 |
| IL-2 | 40.00 | 6.32 | 0.37 | 40.00 | 0.73 | 16.92 | 54.60 |
| IL-4 | 40.00 | 1.66 | 0.37 | 2.31 | 0.73 | 4.43 | 3.15 |
| IL-6 | 40.00 | 2.72 | 0.37 | 10.72 | 0.73 | 7.29 | 14.64 |
| IL-7 | 40.00 | 2.78 | 0.37 | 12.79 | 0.73 | 7.45 | 17.46 |
| IL-8 | 40.00 | 1.99 | 0.37 | 4.46 | 0.73 | 5.32 | 6.09 |
| IL-10 | 40.00 | 4.49 | 0.37 | 19.83 | 0.73 | 12.02 | 27.07 |
| IL-12p70 | 40.00 | 2.33 | 0.37 | 5.09 | 0.73 | 6.22 | 6.94 |
| IL-13 | 40.00 | 6.32 | 0.37 | 40.00 | 0.73 | 16.92 | 54.60 |
| IL-15 | 40.00 | -0.37 | 0.37 | -0.71 | 0.73 | -1.00 | -0.98 |
| IL-16 | 40.00 | 0.18 | 0.37 | -0.35 | 0.73 | 0.48 | -0.48 |
| IL-17 | 40.00 | 1.50 | 0.37 | 2.38 | 0.73 | 4.02 | 3.25 |
| IL-18 | 40.00 | 2.09 | 0.37 | 4.80 | 0.73 | 5.59 | 6.55 |
| IL-21 | 40.00 | 3.65 | 0.37 | 16.70 | 0.73 | 9.75 | 22.80 |
| IL-22 | 40.00 | 0.51 | 0.37 | -0.97 | 0.73 | 1.35 | -1.33 |
| IL-33 | 40.00 | 4.67 | 0.37 | 22.36 | 0.73 | 12.50 | 30.52 |
| Calgranulin | 40.00 | 2.35 | 0.37 | 5.50 | 0.73 | 6.30 | 7.50 |
| Eotaxin | 40.00 | 4.02 | 0.37 | 15.98 | 0.73 | 10.75 | 21.82 |
| GM-CSF | 40.00 | 0.18 | 0.37 | 2.06 | 0.73 | 0.47 | 2.81 |
| Gro-α | 40.00 | 2.39 | 0.37 | 5.81 | 0.73 | 6.40 | 7.94 |
| IFN-γ | 40.00 | 1.03 | 0.37 | 0.57 | 0.73 | 2.77 | 0.78 |
| IP-10 | 40.00 | 1.81 | 0.37 | 2.41 | 0.73 | 4.84 | 3.29 |
| ITAC | 40.00 | 1.77 | 0.37 | 5.34 | 0.73 | 4.74 | 7.29 |
| MCSF | 40.00 | 0.88 | 0.37 | 0.08 | 0.73 | 2.36 | 0.11 |
| MCP-1 | 40.00 | 0.66 | 0.37 | 0.07 | 0.73 | 1.76 | 0.09 |
| MIG | 40.00 | 5.32 | 0.37 | 30.28 | 0.73 | 14.22 | 41.34 |
| MIP-1α | 40.00 | 6.25 | 0.37 | 39.33 | 0.73 | 16.71 | 53.69 |
| MIP-1β | 40.00 | 2.04 | 0.37 | 5.08 | 0.73 | 5.45 | 6.93 |
| MIP-3α | 40.00 | 0.15 | 0.37 | -0.32 | 0.73 | 0.40 | -0.44 |
| TGF-β | 40.00 | 0.96 | 0.37 | 0.77 | 0.73 | 2.58 | 1.06 |
| TNF-α | 40.00 | -0.18 | 0.37 | 0.57 | 0.73 | -0.47 | 0.78 |
| RANTES | 40.00 | 2.01 | 0.37 | 3.73 | 0.73 | 5.37 | 5.10 |
| EBRT T2 Group | | | | | | | |
| EV- associated | | | | | | | |
| T2 BDNF | 40.00 | 0.81 | 0.37 | 1.39 | 0.73 | 2.16 | 1.90 |
| T2 CRP | 40.00 | -0.61 | 0.37 | -0.54 | 0.73 | -1.63 | -0.73 |
| T2 HSP27 | 40.00 | 0.04 | 0.37 | -1.05 | 0.73 | 0.12 | -1.43 |
| T2 HSP70 | 40.00 | 2.54 | 0.37 | 9.22 | 0.73 | 6.79 | 12.58 |
| T2 HSP90 | 40.00 | 4.52 | 0.37 | 22.53 | 0.73 | 12.10 | 30.76 |
| T2 IFNα-2 | 40.00 | 3.50 | 0.37 | 11.20 | 0.73 | 9.35 | 15.29 |
| T2 IL-3 | 40.00 | 5.22 | 0.37 | 29.54 | 0.73 | 13.97 | 40.32 |
| T2 IL-9 | 40.00 | 5.21 | 0.37 | 28.34 | 0.73 | 13.94 | 38.68 |
| T2 IL-6R | 40.00 | 0.30 | 0.37 | -0.05 | 0.73 | 0.82 | -0.07 |
| T2 SDF | 40.00 | -0.03 | 0.37 | 0.00 | 0.73 | -0.08 | 0.01 |
| T2 SURVIVIN | 40.00 | 4.61 | 0.37 | 23.02 | 0.73 | 12.34 | 31.43 |
| T2 TRAIL | 40.00 | 4.19 | 0.37 | 18.97 | 0.73 | 11.22 | 25.90 |
| T2 IL-1α | 40.00 | 6.15 | 0.37 | 38.44 | 0.73 | 16.45 | 52.47 |
| T2 IL-1β | 40.00 | 6.17 | 0.37 | 38.66 | 0.73 | 16.51 | 52.77 |
| T2 IL-2 | 40.00 | 6.32 | 0.37 | 40.00 | 0.73 | 16.92 | 54.60 |
| T2 IL-4 | 40.00 | 6.14 | 0.37 | 38.35 | 0.73 | 16.42 | 52.35 |
| T2 IL-6 | 40.00 | 6.28 | 0.37 | 39.63 | 0.73 | 16.81 | 54.09 |
| T2 IL-7 | 40.00 | 6.19 | 0.37 | 38.76 | 0.73 | 16.55 | 52.90 |
| T2 IL-8 | 40.00 | 6.25 | 0.37 | 39.31 | 0.73 | 16.71 | 53.66 |
| T2 IL-10 | 40.00 | 4.26 | 0.37 | 17.52 | 0.73 | 11.39 | 23.92 |
| T2 IL-12p70 | 40.00 | 6.28 | 0.37 | 39.58 | 0.73 | 16.80 | 54.03 |
| T2 IL-13 | 40.00 | 6.17 | 0.37 | 38.56 | 0.73 | 16.51 | 52.63 |
| T2 IL-15 | 40.00 | 3.55 | 0.37 | 15.43 | 0.73 | 9.49 | 21.06 |
| T2 IL-16 | 40.00 | 6.07 | 0.37 | 37.77 | 0.73 | 16.24 | 51.56 |
| T2 IL-17 | 40.00 | 1.70 | 0.37 | 1.93 | 0.73 | 4.54 | 2.63 |
| T2 IL-18 | 40.00 | 1.74 | 0.37 | 2.94 | 0.73 | 4.66 | 4.01 |
| T2 IL-21 | 40.00 | 6.23 | 0.37 | 39.17 | 0.73 | 16.67 | 53.46 |
| T2 IL-22 | 40.00 | 6.12 | 0.37 | 38.19 | 0.73 | 16.38 | 52.13 |
| T2 IL-33 | 40.00 | 6.07 | 0.37 | 37.55 | 0.73 | 16.23 | 51.25 |
| T2 Calgranulin | 40.00 | 6.32 | 0.37 | 40.00 | 0.73 | 16.92 | 54.60 |
| T2 Eotaxin | 40.00 | 5.89 | 0.37 | 36.07 | 0.73 | 15.75 | 49.23 |
| T2 GM-CSF | 40.00 | 5.14 | 0.37 | 29.70 | 0.73 | 13.76 | 40.54 |
| T2 Gro-α | 40.00 | 3.03 | 0.37 | 11.83 | 0.73 | 8.10 | 16.15 |
| T2 IFN-γ | 40.00 | 6.19 | 0.37 | 38.83 | 0.73 | 16.57 | 53.00 |
| T2 IP-10 | 40.00 | 3.98 | 0.37 | 17.73 | 0.73 | 10.65 | 24.20 |
| T2 ITAC | 40.00 | 3.26 | 0.37 | 15.53 | 0.73 | 8.73 | 21.19 |
| T2 MCSF | 40.00 | 2.32 | 0.37 | 4.89 | 0.73 | 6.19 | 6.68 |
| T2 MCP-1 | 40.00 | 4.16 | 0.37 | 20.95 | 0.73 | 11.13 | 28.60 |
| T2 MIG | 40.00 | 4.53 | 0.37 | 21.90 | 0.73 | 12.12 | 29.90 |
| T2 MIP-1α | 40.00 | 4.89 | 0.37 | 27.71 | 0.73 | 13.08 | 37.83 |
| T2 MIP-1β | 40.00 | 5.84 | 0.37 | 35.60 | 0.73 | 15.61 | 48.59 |
| T2 MIP-3α | 40.00 | 1.12 | 0.37 | 0.47 | 0.73 | 3.00 | 0.64 |
| T2 TGF-β | 40.00 | 6.12 | 0.37 | 38.12 | 0.73 | 16.36 | 52.04 |
| T2 TNF-α | 40.00 | 6.16 | 0.37 | 38.59 | 0.73 | 16.49 | 52.67 |
| T2 RANTES | 40.00 | 2.76 | 0.37 | 9.13 | 0.73 | 7.40 | 12.47 |
| Soluble | | | | | | | |
| T2 BDNF | 40.00 | 3.51 | 0.37 | 15.57 | 0.73 | 9.38 | 21.26 |
| T2 CRP | 40.00 | 2.44 | 0.37 | 8.47 | 0.73 | 6.52 | 11.57 |
| T2 HSP27 | 40.00 | 0.57 | 0.37 | 0.68 | 0.73 | 1.52 | 0.93 |
| T2 HSP70 | 40.00 | 1.31 | 0.37 | 2.82 | 0.73 | 3.50 | 3.85 |
| T2 HSP90 | 40.00 | 0.77 | 0.37 | -0.15 | 0.73 | 2.06 | -0.20 |
| T2 IFNα2 | 40.00 | 4.45 | 0.37 | 22.38 | 0.73 | 11.91 | 30.55 |
| T2 IL-3 | 40.00 | 0.43 | 0.37 | -0.14 | 0.73 | 1.15 | -0.19 |
| T2 IL-9 | 40.00 | 2.31 | 0.37 | 4.59 | 0.73 | 6.17 | 6.26 |
| T2 IL-6R | 40.00 | 2.34 | 0.37 | 7.33 | 0.73 | 6.26 | 10.00 |
| T2 SDF | 40.00 | -0.32 | 0.37 | -1.00 | 0.73 | -0.85 | -1.37 |
| T2 SURVIVIN | 40.00 | 0.86 | 0.37 | -0.20 | 0.73 | 2.29 | -0.28 |
| T2 TRAIL | 40.00 | 1.06 | 0.37 | 1.26 | 0.73 | 2.84 | 1.73 |
| T2 IL-1a | 40.00 | 3.33 | 0.37 | 11.06 | 0.73 | 8.90 | 15.09 |
| T2 IL-1β | 40.00 | 0.96 | 0.37 | -0.66 | 0.73 | 2.58 | -0.90 |
| T2 IL-2 | 40.00 | ND | ND | ND | ND | ND | ND |
| T2 IL-4 | 40.00 | 5.16 | 0.37 | 28.77 | 0.73 | 13.80 | 39.28 |
| T2 IL-6 | 40.00 | 1.74 | 0.37 | 1.64 | 0.73 | 4.65 | 2.24 |
| T2 IL-7 | 40.00 | 0.55 | 0.37 | 0.35 | 0.73 | 1.46 | 0.47 |
| T2 IL-8 | 40.00 | 3.82 | 0.37 | 18.24 | 0.73 | 10.23 | 24.90 |
| T2 IL-10 | 40.00 | 6.32 | 0.37 | 40.00 | 0.73 | 16.92 | 54.60 |
| T2 IL-12p70 | 40.00 | 2.77 | 0.37 | 6.65 | 0.73 | 7.42 | 9.08 |
| T2 IL-13 | 40.00 | ND | ND | ND | ND | ND | ND |
| T2 IL-15 | 40.00 | 0.28 | 0.37 | -1.51 | 0.73 | 0.75 | -2.06 |
| T2 IL-16 | 40.00 | 1.39 | 0.37 | 4.23 | 0.73 | 3.71 | 5.78 |
| T2 IL-17 | 40.00 | 4.01 | 0.37 | 19.14 | 0.73 | 10.72 | 26.13 |
| T2 IL-18 | 40.00 | 1.58 | 0.37 | 2.05 | 0.73 | 4.23 | 2.80 |
| T2 IL-21 | 40.00 | 1.52 | 0.37 | 2.86 | 0.73 | 4.07 | 3.90 |
| T2 IL-22 | 40.00 | 2.31 | 0.37 | 6.35 | 0.73 | 6.18 | 8.67 |
| T2 IL-33 | 40.00 | -3.35 | 0.37 | 9.74 | 0.73 | -8.97 | 13.29 |
| T2 Calg-A | 40.00 | 6.29 | 0.37 | 39.72 | 0.73 | 16.84 | 54.22 |
| T2 Eotaxin | 40.00 | 5.75 | 0.37 | 35.06 | 0.73 | 15.37 | 47.85 |
| T2 GM-CSF | 40.00 | -0.23 | 0.37 | -1.18 | 0.73 | -0.62 | -1.61 |
| T2 Gro-α | 40.00 | 2.95 | 0.37 | 8.59 | 0.73 | 7.89 | 11.73 |
| T2 IFN-γ | 40.00 | 1.89 | 0.37 | 2.34 | 0.73 | 5.06 | 3.19 |
| T2 IP-10 | 40.00 | 2.05 | 0.37 | 3.76 | 0.73 | 5.49 | 5.13 |
| T2 ITAC | 40.00 | 4.95 | 0.37 | 28.14 | 0.73 | 13.24 | 38.41 |
| T2 MCSF | 40.00 | 1.78 | 0.37 | 1.84 | 0.73 | 4.76 | 2.51 |
| T2 MCP-1 | 40.00 | 0.89 | 0.37 | 1.84 | 0.73 | 2.39 | 2.51 |
| T2 MIG | 40.00 | 6.08 | 0.37 | 37.86 | 0.73 | 16.27 | 51.68 |
| T2 MIP-1α | 40.00 | 5.96 | 0.37 | 36.87 | 0.73 | 15.95 | 50.33 |
| T2 MIP-1β | 40.00 | 1.69 | 0.37 | 4.08 | 0.73 | 4.51 | 5.57 |
| T2 MIP3-α | 40.00 | 0.43 | 0.37 | -1.13 | 0.73 | 1.16 | -1.55 |
| T2 TGF-β | 40.00 | 3.65 | 0.37 | 13.76 | 0.73 | 9.77 | 18.78 |
| T2 TNF-α | 40.00 | 0.05 | 0.37 | -1.18 | 0.73 | 0.12 | -1.61 |
| T2 RANTES | 40.00 | 2.14 | 0.37 | 4.33 | 0.73 | 5.73 | 5.91 |
| Active Surveillance Cohort | | | | | | | |
| EV-associated | | | | | | | |
| BDNF | 20.00 | 0.11 | 0.51 | -0.05 | 0.99 | 0.22 | -0.05 |
| CRP | 20.00 | 0.41 | 0.51 | 0.02 | 0.99 | 0.79 | 0.02 |
| HSP27 | 20.00 | 0.08 | 0.51 | -1.02 | 0.99 | 0.16 | -1.03 |
| HSP70 | 20.00 | 2.63 | 0.51 | 6.15 | 0.99 | 5.13 | 6.20 |
| HSP90 | 20.00 | 3.13 | 0.51 | 11.33 | 0.99 | 6.11 | 11.41 |
| IFNα2 | 20.00 | 4.47 | 0.51 | 19.96 | 0.99 | 8.72 | 20.11 |
| IL-3 | 20.00 | 1.59 | 0.51 | 1.31 | 0.99 | 3.10 | 1.32 |
| IL-9 | 20.00 | 2.77 | 0.51 | 6.59 | 0.99 | 5.40 | 6.64 |
| IL-6R | 20.00 | 0.28 | 0.51 | -0.65 | 0.99 | 0.55 | -0.65 |
| SDF | 20.00 | 0.03 | 0.51 | -0.61 | 0.99 | 0.05 | -0.61 |
| Survivin | 20.00 | 1.79 | 0.51 | 4.01 | 0.99 | 3.49 | 4.04 |
| TRAIL | 20.00 | 2.32 | 0.51 | 4.77 | 0.99 | 4.52 | 4.81 |
| IL-1α | 20.00 | 4.32 | 0.51 | 19.06 | 0.99 | 8.44 | 19.21 |
| IL-1β | 20.00 | 3.02 | 0.51 | 11.45 | 0.99 | 5.89 | 11.53 |
| IL-2 | 20.00 | 4.39 | 0.51 | 19.47 | 0.99 | 8.58 | 19.62 |
| IL-4 | 20.00 | 4.08 | 0.51 | 17.43 | 0.99 | 7.97 | 17.56 |
| IL-6 | 20.00 | 2.97 | 0.51 | 8.60 | 0.99 | 5.80 | 8.67 |
| IL-7 | 20.00 | 3.45 | 0.51 | 13.87 | 0.99 | 6.74 | 13.97 |
| IL-8 | 20.00 | 4.03 | 0.51 | 16.94 | 0.99 | 7.86 | 17.07 |
| IL-10 | 20.00 | 4.46 | 0.51 | 19.90 | 0.99 | 8.70 | 20.05 |
| IL-12p70 | 20.00 | 3.39 | 0.51 | 11.94 | 0.99 | 6.62 | 12.03 |
| IL-13 | 20.00 | 4.46 | 0.51 | 19.91 | 0.99 | 8.71 | 20.07 |
| IL-15 | 20.00 | 3.18 | 0.51 | 11.66 | 0.99 | 6.21 | 11.75 |
| IL-16 | 20.00 | 1.45 | 0.51 | 1.72 | 0.99 | 2.83 | 1.74 |
| IL-17 | 20.00 | 3.24 | 0.51 | 11.81 | 0.99 | 6.33 | 11.90 |
| IL-18 | 20.00 | 1.18 | 0.51 | 0.03 | 0.99 | 2.31 | 0.03 |
| IL-21 | 20.00 | 3.85 | 0.51 | 15.90 | 0.99 | 7.52 | 16.02 |
| IL-22 | 20.00 | 1.57 | 0.51 | 3.03 | 0.99 | 3.06 | 3.06 |
| IL-33 | 20.00 | 2.95 | 0.51 | 7.62 | 0.99 | 5.77 | 7.68 |
| Calgranulin | 20.00 | ND | ND | ND | ND | ND | ND |
| Eotaxin | 20.00 | 2.96 | 0.51 | 9.36 | 0.99 | 5.78 | 9.43 |
| GM-CSF | 20.00 | 2.89 | 0.51 | 10.56 | 0.99 | 5.65 | 10.65 |
| Gro-α | 20.00 | 3.42 | 0.51 | 13.56 | 0.99 | 6.68 | 13.66 |
| IFN-γ | 20.00 | 4.09 | 0.51 | 17.62 | 0.99 | 7.98 | 17.76 |
| IP-10 | 20.00 | 2.40 | 0.51 | 5.47 | 0.99 | 4.69 | 5.51 |
| ITAC | 20.00 | 3.42 | 0.51 | 13.66 | 0.99 | 6.68 | 13.77 |
| MCSF | 20.00 | 4.02 | 0.51 | 17.07 | 0.99 | 7.85 | 17.20 |
| MCP-1 | 20.00 | 3.13 | 0.51 | 10.36 | 0.99 | 6.12 | 10.44 |
| MIG | 20.00 | 0.91 | 0.51 | -0.27 | 0.99 | 1.78 | -0.27 |
| MIP-1α | 20.00 | 3.27 | 0.51 | 12.76 | 0.99 | 6.39 | 12.86 |
| MIP-1β | 20.00 | 2.61 | 0.51 | 8.05 | 0.99 | 5.10 | 8.11 |
| MIP-3α | 20.00 | 2.24 | 0.51 | 6.88 | 0.99 | 4.38 | 6.94 |
| TGF-β | 20.00 | 3.39 | 0.51 | 12.62 | 0.99 | 6.63 | 12.72 |
| TNF-α | 20.00 | 3.31 | 0.51 | 12.90 | 0.99 | 6.47 | 13.00 |
| RANTES | 20.00 | 1.71 | 0.51 | 3.08 | 0.99 | 3.35 | 3.10 |
| Soluble | | | | | | | |
| BDNF | 20.00 | 2.10 | 0.51 | 4.42 | 0.99 | 4.09 | 4.45 |
| CRP | 20.00 | 2.45 | 0.51 | 5.15 | 0.99 | 4.79 | 5.18 |
| HSP27 | 20.00 | 0.54 | 0.51 | -1.25 | 0.99 | 1.06 | -1.26 |
| HSP70 | 20.00 | 0.58 | 0.51 | 0.21 | 0.99 | 1.13 | 0.21 |
| HSP90 | 20.00 | 0.98 | 0.51 | 0.43 | 0.99 | 1.91 | 0.44 |
| IFNα-2 | 20.00 | 4.45 | 0.51 | 19.87 | 0.99 | 8.69 | 20.02 |
| IL-3 | 20.00 | 0.81 | 0.51 | -0.76 | 0.99 | 1.59 | -0.76 |
| IL-9 | 20.00 | 2.21 | 0.51 | 4.37 | 0.99 | 4.31 | 4.40 |
| IL-6R | 20.00 | 1.67 | 0.51 | 2.95 | 0.99 | 3.26 | 2.98 |
| SDF | 20.00 | -0.10 | 0.51 | -1.06 | 0.99 | -0.19 | -1.07 |
| SURVIVIN | 20.00 | 0.70 | 0.51 | -0.81 | 0.99 | 1.38 | -0.82 |
| TRAIL | 20.00 | 0.22 | 0.51 | -1.12 | 0.99 | 0.44 | -1.13 |
| IL-1α | 20.00 | 2.80 | 0.51 | 9.67 | 0.99 | 5.46 | 9.74 |
| IL-1β | 20.00 | -0.31 | 0.51 | -0.11 | 0.99 | -0.61 | -0.11 |
| IL-2 | 20.00 | 4.47 | 0.51 | 20.00 | 0.99 | 8.73 | 20.15 |
| IL-4 | 20.00 | 0.78 | 0.51 | 1.93 | 0.99 | 1.52 | 1.94 |
| IL-6 | 20.00 | 1.68 | 0.51 | 2.65 | 0.99 | 3.28 | 2.67 |
| IL-7 | 20.00 | 2.24 | 0.51 | 6.84 | 0.99 | 4.38 | 6.90 |
| IL-8 | 20.00 | 1.98 | 0.51 | 4.94 | 0.99 | 3.86 | 4.97 |
| IL-10 | 20.00 | 2.24 | 0.51 | 3.61 | 0.99 | 4.37 | 3.64 |
| IL-12p70 | 20.00 | 1.26 | 0.51 | 0.00 | 0.99 | 2.46 | 0.00 |
| IL-13 | 20.00 | 4.47 | 0.51 | 20.00 | 0.99 | 8.73 | 20.15 |
| IL-15 | 20.00 | 0.61 | 0.51 | 1.10 | 0.99 | 1.19 | 1.11 |
| IL-16 | 20.00 | 2.76 | 0.51 | 10.01 | 0.99 | 5.40 | 10.09 |
| IL-17 | 20.00 | 0.68 | 0.51 | -0.37 | 0.99 | 1.33 | -0.37 |
| IL-18 | 20.00 | 1.34 | 0.51 | 1.02 | 0.99 | 2.62 | 1.02 |
| IL-21 | 20.00 | 2.08 | 0.51 | 7.04 | 0.99 | 4.07 | 7.09 |
| IL-22 | 20.00 | -0.13 | 0.51 | -0.83 | 0.99 | -0.26 | -0.83 |
| IL-33 | 20.00 | 2.88 | 0.51 | 7.08 | 0.99 | 5.63 | 7.13 |
| Calgranulin | 20.00 | 4.03 | 0.51 | 16.89 | 0.99 | 7.87 | 17.02 |
| Eotaxin | 20.00 | -1.05 | 0.51 | 1.38 | 0.99 | -2.06 | 1.39 |
| GM-CSF | 20.00 | 1.64 | 0.51 | 3.01 | 0.99 | 3.20 | 3.03 |
| Gro-α | 20.00 | 1.32 | 0.51 | 1.17 | 0.99 | 2.57 | 1.18 |
| IFN-γ | 20.00 | -0.01 | 0.51 | 0.69 | 0.99 | -0.02 | 0.69 |
| IP-10 | 20.00 | 1.45 | 0.51 | 1.09 | 0.99 | 2.84 | 1.09 |
| ITAC | 20.00 | -0.22 | 0.51 | -0.73 | 0.99 | -0.42 | -0.74 |
| MCSF | 20.00 | 0.54 | 0.51 | 0.47 | 0.99 | 1.05 | 0.47 |
| MCP-1 | 20.00 | 1.79 | 0.51 | 4.21 | 0.99 | 3.49 | 4.24 |
| MIG | 20.00 | 1.39 | 0.51 | 0.86 | 0.99 | 2.72 | 0.86 |
| MIP-1α | 20.00 | 1.33 | 0.51 | 2.74 | 0.99 | 2.61 | 2.76 |
| MIP-1β | 20.00 | 2.70 | 0.51 | 9.56 | 0.99 | 5.27 | 9.63 |
| MIP-3α | 20.00 | 0.44 | 0.51 | 0.35 | 0.99 | 0.86 | 0.36 |
| TGF-β | 20.00 | 0.29 | 0.51 | -1.00 | 0.99 | 0.57 | -1.01 |
| TNF-α | 20.00 | 0.79 | 0.51 | 1.51 | 0.99 | 1.54 | 1.52 |
| RANTES | 20.00 | 1.29 | 0.51 | 2.29 | 0.99 | 2.52 | 2.31 |

**Abbreviations:** BDNF = Brain-Derived Neurotrophic Factor, CRP = C-Reactive Protein, HSP = Heat Shock Protein, IFN = Interferon, SDF = Stromal Derived Factor, TRAIL = TNF-related apoptosis-inducing ligand, GM-CSF = Granulocyte Macrophage-Colony Stimulating Factor, Gro-α = Growth regulated alpha, IP-10 = Interferon-γ-induced protein, ITAC = Inducible T-cell alpha chemoattractant, M-CSF = Macrophage-Colony Stimulating Factor, MCP-1 = Monocyte Chemoattractant Protein-1, MIG = Monokine induced by IFN- γ, RANTES = Regulated on Activation, Normal T Expressed and Secreted, ND=not detected.
